# Stromal cell-derived factor (SDF)-1α and platelet-rich plasma enhance bone regeneration and angiogenesis simultaneously in situ in rabbit calvaria

**DOI:** 10.1007/s10856-021-06600-z

**Published:** 2021-09-15

**Authors:** Zhengye Zhang, Yang Zheng, Jianing Zu, Jinpeng Zhuang, Gongping Xu, Jinglong Yan, Xiaoqi Liu

**Affiliations:** Department of Orthopedic Surgery, The Second Affiliated Hospital, Harbin Medical University, Harbin, 150001 PR China

## Abstract

The current study aimed to evaluate the effects of chemokine stromal cell-derived factor (SDF)-1α and platelet-rich plasma (PRP) on bone formation and angiogenesis, and to assess whether SDF-1α and PRP could function synergistically. Four evenly distributed defects (8 mm in diameter) were generated in the calvarial bones of New Zealand white rabbits. All rabbits received four treatment regimens containing autogenous bone particles (AB), SDF-1α, or PRP. AB group presented significantly less bone formation compared with the other three groups 2 and 4 weeks after surgery. The amount of newly formed bone in the AB+PRP+SDF-1α group was similar to that in the AB + SDF-1α group at the 4-week time-point (*p* = 0.65), and was much greater than that in the AB and AB+PRP group (*p* < 0.001). Meanwhile, more new blood vessels were formed in the AB+PRP, AB+SDF-1α, and AB+PRP+SDF-1α group versus the AB group. AB+PRP+SDF-1α group showed statistically increased angiogenesis compared with the AB+PRP and AB+SDF-1α groups (both *p* < 0.05) after treatment for 2 and 4 weeks. These findings indicated that SDF-1α and PRP might exhibit synergistic effects to promote angiogenesis in early bone regeneration.

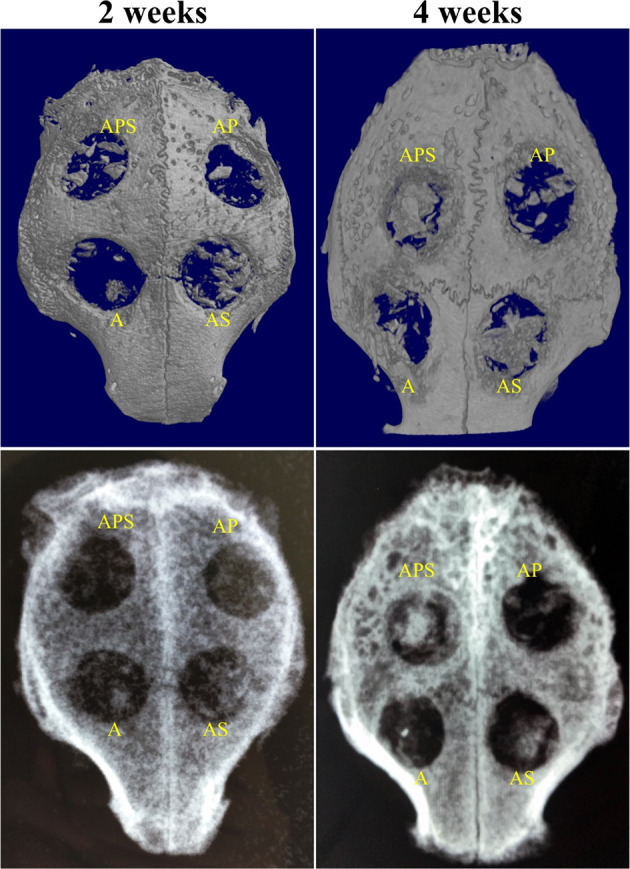

## Introduction

Bone defects remain one of the most common clinical challenges around the world, which could be caused by tumor resection, trauma, or infection [1–3]. Conventional approaches in tissue engineering based on seeding of bone marrow mesenchymal stem cells (MSCs) have achieved successful effects [4–6]. However, host vascular system destruction and hematoma at the site of fracture may lead to death or functional deficits of the inoculated bone marrow MSCs.

SDF-1α or CXCL12 represents a chemotactic protein, which interacts with G-protein-coupled receptors [7]. Although SDF-1α is designated as a CXC chemokine, it is homologous to other CC and CXC chemokines, with 22% and 27% sequence similarity, respectively [8]. SDF-1α is intrinsically linked to bone and cartilage physiologies. For instance, osteoblast cells secrete SDF-1α upon induction by multiple cytokines such as interleukin-1β [9], tumor necrosis factor-alpha, and transforming growth factor-β (TGF-β). Furthermore, articular chondrocytes express CXCR4 receptor and produce matrix metalloproteinases (MMP)-3, 9, and 13 in response to SDF-1α secretion [10]. Notably, other chondrocyte populations, including epiphyseal hypertrophic chondrocytes, produce CXCR4 and MMP-13 [11].

In addition, the role of platelet-rich plasma (PRP) in bone repair remains controversial [12–15]. PRP may exert a beneficial effect on osseous regeneration [16]. Meanwhile, other studies reported that early and late bone healing processes were not enhanced by PRP [17–19]. PRP did not provide any statistically significant benefit in the management of rabbit mandibular defects [20]. Furthermore, high amount of PRP alone or in combination with bone graft particles can prevent the repair of non-critical defects in rabbit calvaria [21]. Therefore, we hypothesized that SDF-1α and PRP could exert synergistic effects on angiogenesis.

## Materials and methods

### Animals

Sixteen 6-month-old New Zealand white rabbits weighing about 3 kg were used in the present study. All rabbits were treated humanely in accordance with The Code of Ethics of the World Medical Association for animal experiments (Revision of Directive 86/609/EEC). The study was approved by the ethics committee of our institution.

### Preparation of PRP and AB particles

Prior to the induction of calvarial bone defects (CBD), 40 ml blood was collected from each rabbit by cardiac puncture with citrate anticoagulation. Intravenous saline (40 ml) injection was performed to maintain systemic blood volume.

Erythrocytes in whole blood sample were separated from leukocytes, platelets, and plasma by centrifugation at 1500 rpm for 20 min. The upper layer of the preparation contained platelets and leukocytes, while the lower layer comprised of red blood cells. All the supernatant and the upper 1 to 2 mm layer of erythrocytes were transferred into a new tube, and centrifuged at 3000 rpm for 20 min. Then the platelets, leukocytes, and the remaining erythrocytes were separated and precipitated from the plasma. Subsequently, approximately three quarters of the supernatant was discarded, and the remaining 0.18 ml contained PRP. Whole blood and PRP preparations were diluted 1:100 in Brecher’s solution for erythrocyte lysis. Then, the platelets in the diluted whole blood and PRP samples were assessed using a Neubauer chamber. All procedures were performed under sterile conditions [22]. The PRP preparation was inoculated into surgical defects after 45 min upon activation with calcium chloride (0.5% final concentration). A bone mill was used to prepare AB from calvarial bone during the creation of the surgical defects. Calvarial bone was grinded using a spherical grinding drill in saline. Particles of 210–270 μm were isolated using a sub-sieve sizer and centrifuged at 120 rpm for 5 min. Approximately 0.2 ml or 0.1 ml of AB added with PRP preparation was immediately introduced into the surgical defects.

### Surgical procedure

**Fig. 1:**
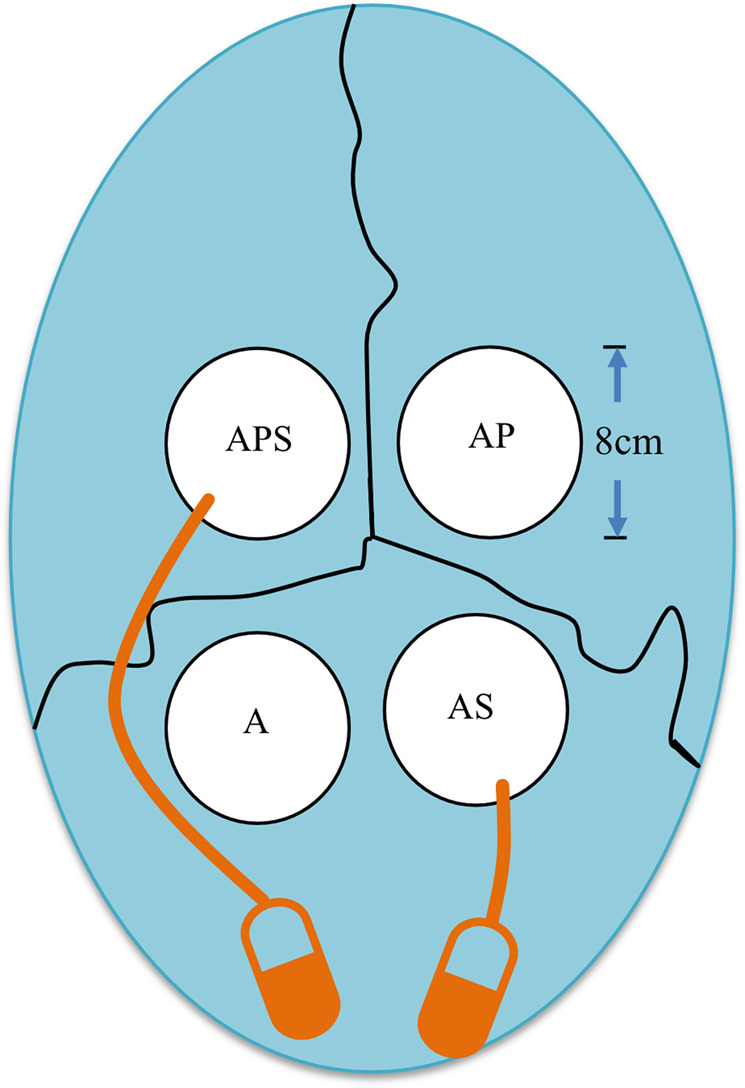
Diagram showing the four evenly distributed surgical defects (8 mm diameter), allocated in the calvarial bones of each rabbit. A group, autogenous bone particles alone (AB); AS group, AB+SDF-1α; AP group, AB+PRP; APS group, AB+PRP+SDF-1α. Orange pumps: two micro-osmotic pumps for loading recombinant human SDF-1α

After the rabbits were anesthetized under aseptic conditions, semilunar scalp incisions were made on the anterior skull to ensure the removal of the full-thickness flaps in the posterior direction. While continuously irrigating with sterile saline, four skull CBD (8 mm in diameter) were generated with a trephine used in low-speed handpiece (Fig. 1) without damaging the dura, flushing the operative areas as described previously [23]. Four treatment regimens were administered to each rabbit: (1) autogenous bone particles alone (AB) (0.2 ml) (group A); (2) AB (0.2 ml)+PRP (AP group); (3) AB (0.2 ml)+SDF-1α (AS group); (4) AB (0.1 ml)+PRP+SDF-1α (APS group). Two 2005 micro-osmotic pumps (Durect, USA) were implanted into two of the four CBDs in the calvaria of each rabbit. All rabbits were subjected to two osmotic pumps containing recombinant human SDF-1α (PeproTech, USA) in PBS (250 µg/ml). The infusion amount of SDF-1α in the AS and APS groups was 3.6 µg per day, for a total of 25 µg. The osmotic pumps were placed on the inner skin of the head. After careful filling, the surgeons were blinded to the treatment regimens. Soft tissue was closed with interrupted sutures. Each rabbit was injected intramuscularly with 24,000 IU of penicillin G-benzathine for three consecutive days after the operation.

### Histological preparation

At 2- and 4-week after surgery, an En bloc removal procedure was conducted on the initial defect areas and neighboring tissues. Head X-ray and micro-CT examinations were carried out. Calvarial specimens were fixed in 10% formalin for 24 h and decalcified in 10% ethylenediamine tetra acetic acid solution for 14 days. Then the specimens were divided into equal parts along the longitudinal direction, dehydrated by graded ethanol, cleared in xylene, and embedded in paraffin. A series of 5 μm-thick vertical sections were obtained from the middle of each defect and Masson’s Trichrome staining was performed. The central portion of each defect was identified and subjected to histologic and histometric analysis. On average, three central sections were used for histologic evaluation, representing the central portion of the osteotomy defect.

### Radiographic analysis

Rabbits underwent euthanasia by anesthetics overdose at 14 and 28 days after operation, respectively. The calvarium bone was then harvested, and fixed in 4% formalin for 48 h. Standard anteroposterior radiographs were taken for bone formation assessment. A Micro*-*CT instrument (Scanco Medical, Switzerland) was utilized to examine the specimens (spatial resolution, 15 µm; 500 projections, 180°; aluminum filter, 1 mm; 100 kV, 100 mA). Three-dimensional image reconstruction was performed using the VGS Studio Max software. Image analysis was standardized according to the method reported by Jung et al. [23]. The formula was as follows: New bone area ratio (%) = Number of pixels of new bone area/Total number of pixels of the defect × 100%.

### Immunohistochemistry

Immunohistochemical analysis was conducted on the specimens with bone formation detected by histology. After incubating with the anti-CD34 primary antibody (Abcam, UK, 1:500), the secondary antibody was added. StreptABComplex/HRP (Dako Corp., USA) was then employed to amplify the signals of cells labeled with diaminobenzidine (Dako, USA).

### Statistical analysis

SPSS 18.0 (SPSS, USA) was used to perform the statistical analysis. The degree of bone formation and the number of blood vessels were determined in the previously defective area. The values were represented as mean ± standard deviation. One-way analysis of variance with post hoc Tukey’s test was adopted for comparisons. A *p* value of <0.05 indicated statistical significance.

## Results

### Platelet counts

The platelet count in PRP preparation (2264.19 ± 372.52 × 10^3^/µl) was increased by 5.4-fold compared to that in whole blood sample (414.20 ± 66.37 × 10^3^/µl).

### Histological findings

**Fig. 2:**
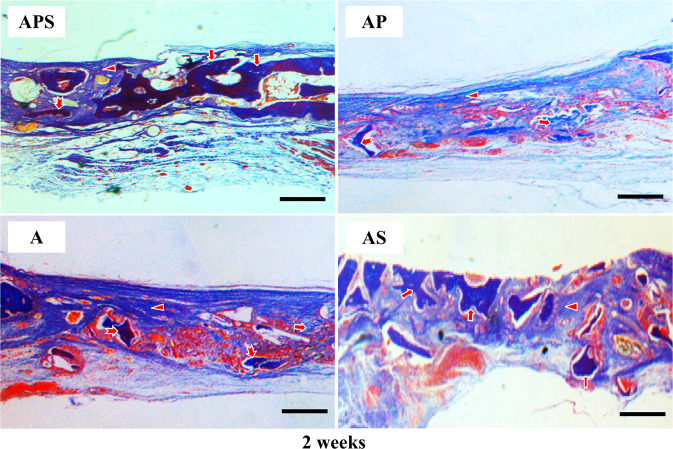
Histological analysis of defects at 2 weeks (Masson’s Trichrome staining). A group, autogenous bone particles alone (AB); AS group, AB+SDF-1α; AP group, AB+PRP; APS group, AB+PRP+SDF-1α. Original magnification, ×100. Arrow head, collagen fibers; arrow, new bone formation; swallow-tailed arrow, residual bone graft. Bar size, 200 μm

**Fig. 3:**
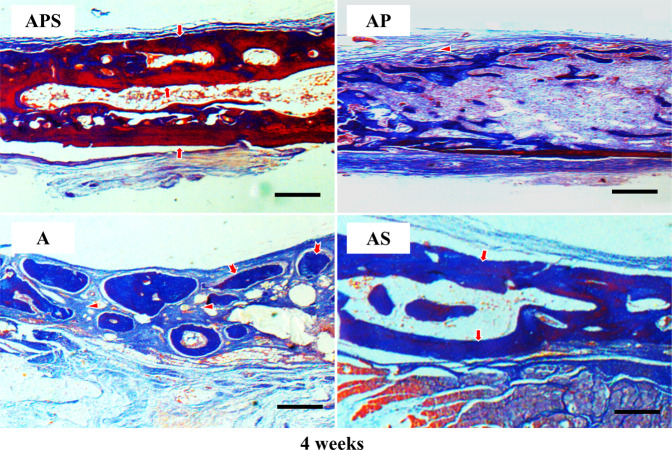
Histological analysis of defects at 4 weeks (Masson’s Trichrome staining). A group, autogenous bone particles alone (AB); AS group, AB+SDF-1α; AP group, AB+PRP; APS group, AB+PRP+SDF-1α. Original magnification, ×100. Arrow head, collagen fibers; arrow, new bone formation; swallow-tailed arrow, residual bone graft. Bar size, 200 μm

**Fig. 4:**
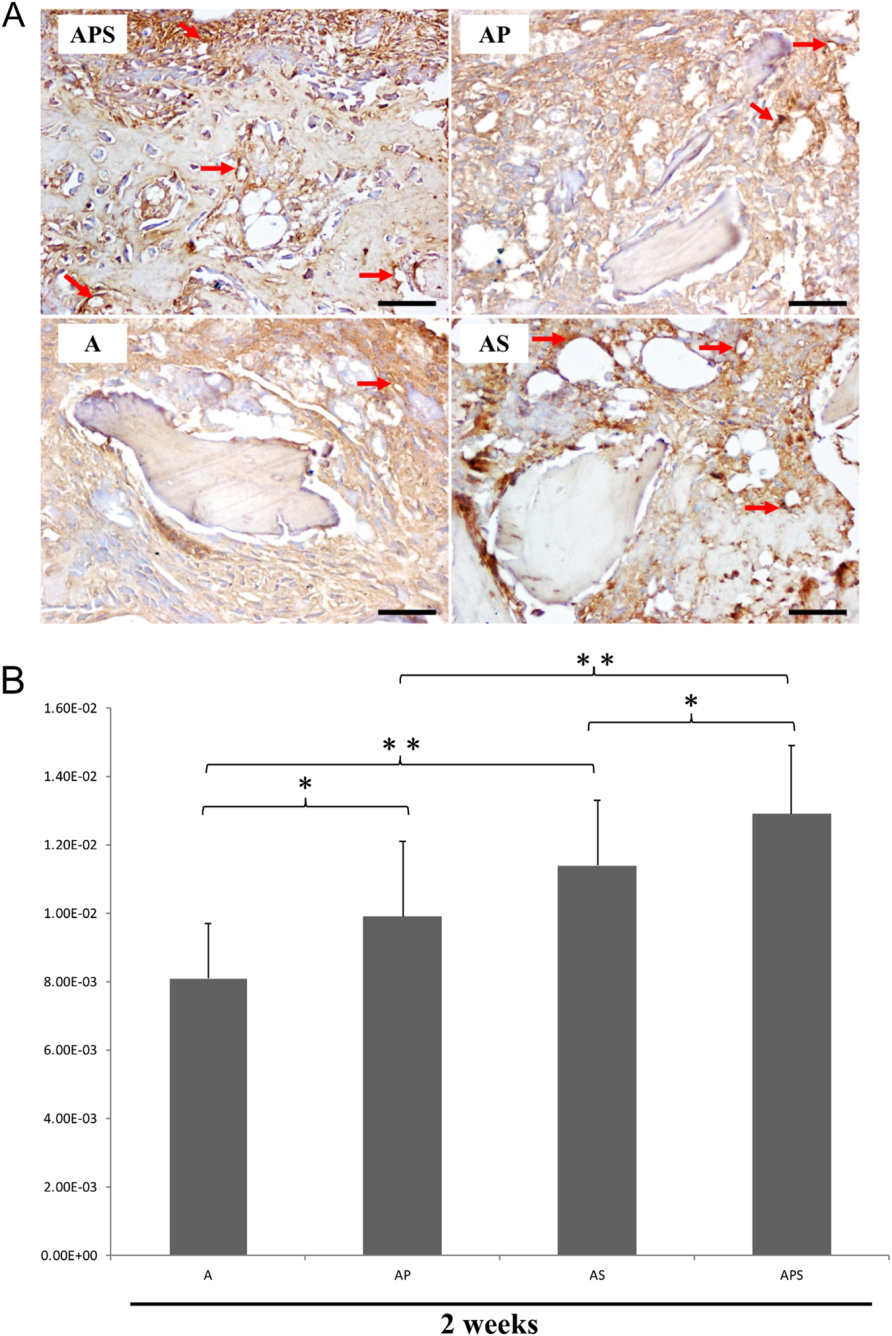
**A** Immunohistochemical detection of CD34 revealing neovascularization in the A, AP, AS, and APS groups at 2 weeks. Original magnification, ×400. Arrow, nascent blood vessels. Bar size, 50 μm. **B** Quantitative analysis of CD34-dependent vessel density in four groups by the IPP software. *p < 0.05, **p < 0.01

At 2-week postoperatively, the connective tissue containing collagen fibers entirely covered the defects in Group A. A limited number of new bone cells were found near the edge of the initial defect (Fig. 2), and most of the implanted bone particles were not resorbed (Supplementary Fig. 1). Furthermore, a small quantity of new bone was found close to the defect center 4 weeks after operation (Fig. 3). In a few samples, no new bone generation from bone graft particles was shown. A small number of newly formed blood vessels were observed in the tissue. In the AP group, all defective areas showed the presence of residual bone graft particles and multiple collagen fibers. At 2-week after surgery, nascent bone was found at the initial boundary and center of each defect (Fig. 2). A few small blood vessels were also observed at the edge of the defects (Fig. 4). After 4 weeks, abundant mineralized bone was found in the defect center (Fig. 3). Near-complete bone defect reconstruction was observed in 16 samples of the AS group after 2 and 4 weeks (Figs. 2 and 3). The AS group showed larger new bone areas in comparison with the other three groups. The area of nascent bone in the APS group was comparable to the AP group at 2-week, and similar to that in the APS group at 4-week postoperatively (Table 1). Neovascularization was prominent 2 weeks after operation (Fig. 4). In the AP and APS groups, the majority of bone graft particles regenerated nascent bone in their surroundings. Residual bone grafts were found throughout the entire defect in almost all AS group specimens after 2 and 4 weeks (Figs. 2 and 3). In the APS group, the majority of specimens resorbed large bone grafts after 4 weeks (Figs. 2 and 3, Supplementary Fig. 1).

**Table 1:** New bone rates in defects

	A	AP	AS	APS
2 weeks				
* n*	8	8	8	8
Mean (%)	5.77	^a^8.04	^b^18.26	^c^8.70
SD (%)	0.77	1.51	3.83	1.14
4 weeks				
*n*	8	8	8	8
Mean (%)	16.58	^d^23.46	^e^40.11	^f^38.85
SD (%)	3.76	4.63	6.26	6.85

A = AB; AP = AB+PRP; AS = AB+SDF-1α; APS = AB+PRP+SDF-1α

*AB* autogenous bone particles alone, *PRP* platelet-rich plasma, SDF-1α stromal cell-derived factor-1α

2 weeks; ^a^versus A (*p* < 0.05); ^b^versus A, AP, and APS (*p* < 0.001); ^c^versus A (*p* < 0.05); 4 weeks; ^d^versus A (*p* < 0.05); ^e^versus A and AP (*p* < 0.001); ^f^versus A and AP (*p* < 0.01); ^f^versus AS (*p* > 0.05); *p* < 0.05 indicated statistical significance

**Fig. 5:**
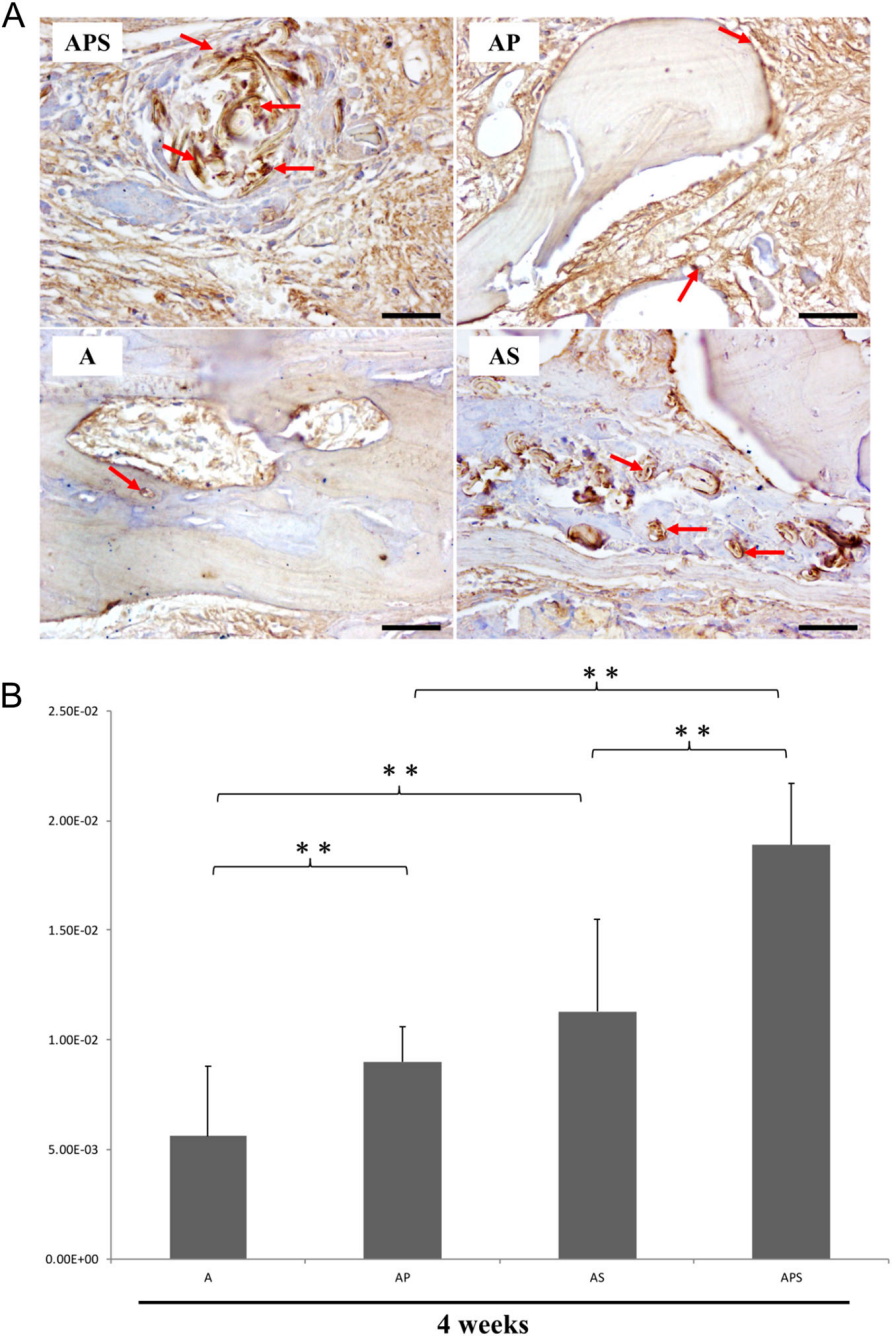
**A** Immunohistochemical detection of CD34 revealing neovascularization in the A, AP, AS, and APS groups at 4 weeks. Original magnification, ×400. Arrow, nascent blood vessels. Bar size, 50 μm. **B** Quantitative analysis of CD34-dependent vessel density in four groups by the IPP software. *p < 0.05, **p < 0.01, Supplementary Fig. 1. Anteroposterior radiographs showing the status of bone regeneration in the four groups. Left, 2 weeks postoperative. Right, 4 weeks postoperative. The upper section is CT 3D reconstruction images, and the lower section is X-ray images

At 2-week following surgery, blood vessel formation was less evident in group A. AS group showed almost two-fold increase in the number of vessels compared to group A. The number of nascent vessels in the APS group was higher than that in the AS and AP group (Figs. 4 and 5).

### Imaging analysis

No inflammation and infection were detected in the collected samples. Bone formation was examined by micro-CT. At postoperative week 2, the area fraction of nascent bone in surgical defects was about 5.77 ± 0.77% in the A group, 8.04 ± 1.51% in the AP group, 18.26 ± 3.83% in the AS group, and 8.70 ± 1.14% in the APS group (Table 1). Bone formation significantly differed between A and AP groups (*p* = 0.045). AS group showed markedly elevated bone generation compared with A and AP groups (*p* < 0.001). AS and APS groups showed comparable osteogenesis values (*p* = 0.54) (Table 1). At week 4, the new bone area fraction in surgical defects were 16.58 ± 3.76% in the A group, 23.46 ± 4.63% in the AP group, 40.11 ± 6.26% in the AS group, and 38.85 ± 8.65% in the APS group (Table 1). No significant difference was observed between the AS and APS groups (*p* = 0.65), whereas there was a marked difference between the AP and APS groups (*p* < 0.001). It was noted that bone generation was centripetal, progressing from the edge toward the center.

## Discussion

In this study, SDF-1α was used to recruit autologous bone MSCs to defect areas for enhancing nascent bone formation. The application of SDF-1α in tissue regeneration is hampered by its shortened half-life and time-dependent delivery to the target injury sites. Therefore, a micro-osmotic pump was employed for continuous SDF-1α delivery and recruitment of progenitor cells. The results indicated increased bone generation in the AS group compared with A group at postoperative weeks 2 and 4 (*p* < 0.05).

It has been reported that the SDF-1α-CXCR4 pathway might contribute to MSC recruitment to the injured tissue [24]. Meanwhile, increased autologous stem cell recruitment improves the tissue response and accelerates stem cell differentiation during the healing process [25]. In conjunction with previous data involving MSC recruitment and differentiation, an in situ regenerative medicine study based on targeted delivery of MSCs to degenerated tissue sites suggested that SDF-1α was a promising therapeutic candidate [26].

We investigated the relationship between vessel density and new bone formation in all groups 2 weeks postoperatively. Abundant small blood vessels were observed in the newly reconstructed surgical defects 2 weeks after SDF-1α infusion. Interestingly, both SDF-1α protein and gene delivered to ischemic tissues enhanced endothelial progenitor cell (EPC) recruitment to ischemic muscles, which in turn promoted angiogenesis [27–30]. In addition, cytokines that induce angiogenesis, including vascular endothelial growth factor (VEGF), fibroblast growth factor (FGF), and hepatocyte growth factor, are up-regulated by SDF-1α [28, 29]. Increased VEGF induces or triggers angiogenic differentiation of stem cells, and even contributes to new vessel formation via mobilization of bone marrow-derived EPCs [31]. In this study, a significant difference in vessel density between SDF-1α-treatment and non-SDF-1α-treatment groups was found. SDF-1α generated a pro-angiogenic environment to accelerate bone formation.

Meanwhile, PRP containing elevated amounts of growth factors (GFs) releases several GFs in the fracture rim, including platelet-derived growth factor (PDGF), TGF-β, FGF, and VEGF [32]. Previous reports demonstrated that TGF-β and PDGF induce osteogenesis in animal models [33, 34], while VEGF enhances bone generation and healing by improving vessel formation [35]. Therefore, PRP supplementation is a promising candidate for bone healing.

Based on our results, the vessel density was different between the AS and APS groups. PRP had a positive effect on neovascularization. Combination of PRP with SDF-1α resulted in increased angiogenesis and vertical bone augmentation [36] in rabbit calvaria. It significantly improved bone healing and bone formation [37, 38]. The effects may be attributed to the role of GFs released from the activated platelets. These GFs induced neovascularization and chemotaxis of tenocytes, and stimulated fibroblast and tenocyte proliferation, and collagen synthesis. Autologous PRP simultaneously increased the expression of several GFs and subsequently enhanced bone healing.

Overall, this study demonstrated that SDF-1α potentially enhanced the area of newly formed bone within the engineered defects during the early stage of bone healing. Furthermore, application of SDF-1α avoided several notable limitations in MSCs utilization. In contrast to exogenous MSCs, SDF-1α is a promising candidate for in situ regenerative therapies. The combination of SDF-1α and PRP may have a synergistic effect on bone formation in surgically induced bone defects.

To the best of our knowledge, our study demonstrated the role of SDF-1α in bone regeneration in vivo. However, the exact mechanism underlying bone regeneration by SDF-1α was still unclear. Further studies are needed to clarify the mechanisms before clinical application.

## Conclusions

Histologic and histomorphometric analyses after 2 and 4 weeks of treatment indicated that both SDF-1α and PRP were promising candidates for accelerated bone regeneration. SDF-1α combined with PRP showed synergistic effects on angiogenesis during the early phase of bone healing.

## Supplementary information

Supplementary figure
