# Supplementary figures and images for: Stromal cell-derived factor (SDF)-1α and platelet-rich plasma enhance bone regeneration and angiogenesis simultaneously in situ in rabbit calvaria
Source: J Mater Sci Mater Med. 2021 Sep 15;32(9):125. doi: 10.1007/s10856-021-06600-z (PMC8443516; doi:10.1007/s10856-021-06600-z)

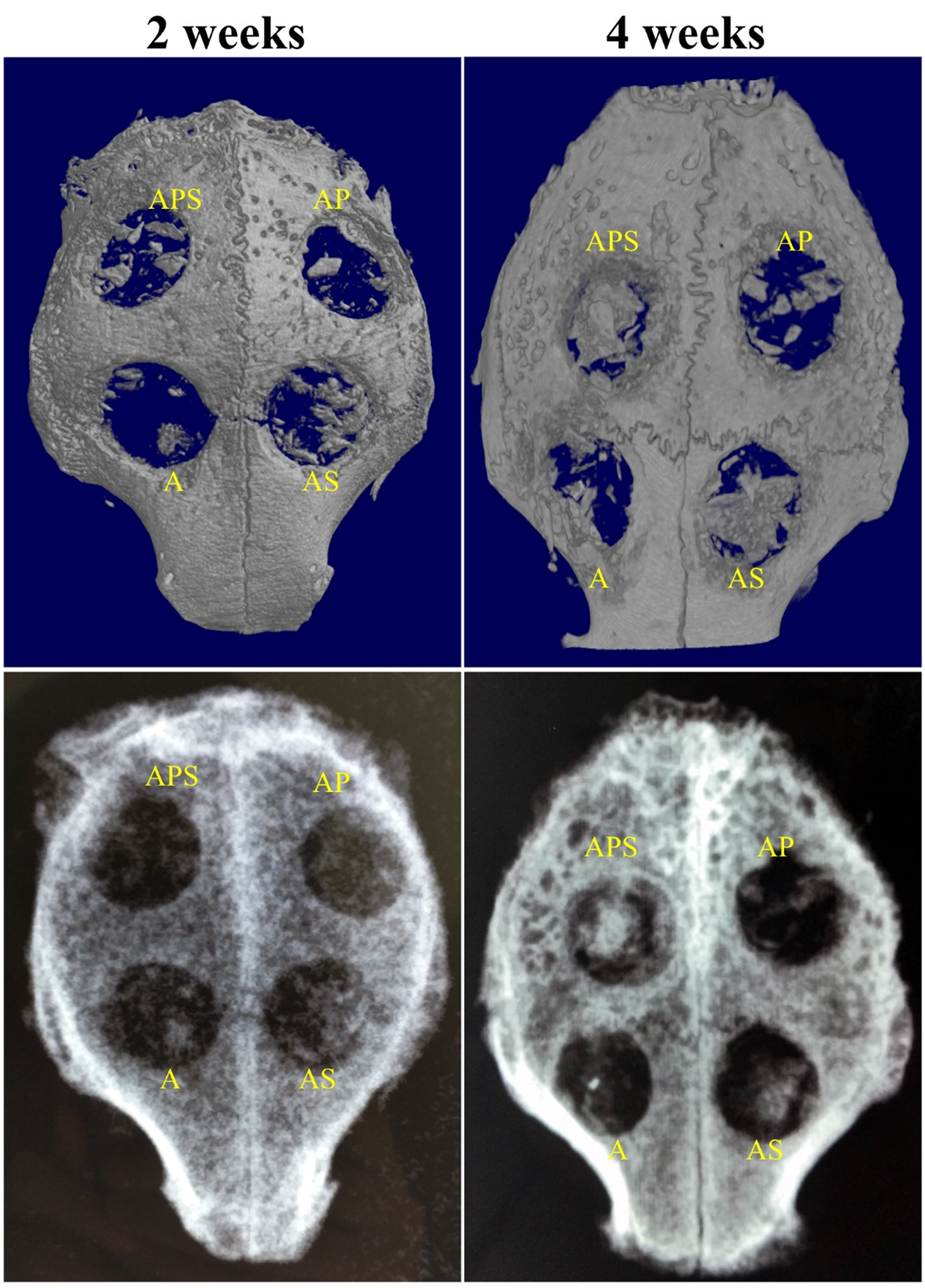

Supplement: Supplementary file 1 — Supplementary figure [file 10856_2021_6600_MOESM1_ESM.tif]
